# Antibacterial Bio-Nanocomposite Textile Material Produced from Natural Resources

**DOI:** 10.3390/nano12152539

**Published:** 2022-07-24

**Authors:** Darka Marković, Andrea Zille, Ana Isabel Ribeiro, Daiva Mikučioniene, Barbara Simončič, Brigita Tomšič, Maja Radetić

**Affiliations:** 1 Innovation Centre of the Faculty of Technology and Metallurgy, University of Belgrade, Karnegijeva 4, 11120 Belgrade, Serbia; 2 Centro de Ciência e Tecnologia Têxtil (2C2T), Universidade do Minho, 4800-058 Guimarães, Portugal; azille@det.uminho.pt (A.Z.); afr@2c2t.uminho.pt (A.I.R.); 3 Faculty of Mechanical Engineering and Design, Kaunas University of Technology, Studentu Str. 56-249, LT-51424 Kaunas, Lithuania; daiva.mikucioniene@ktu.lt; 4 Faculty of Natural Sciences and Engineering Ljubljana, University of Ljubljana, Aškerčeva cesta 12, 1000 Ljubljana, Slovenia; barbara.simoncic@ntf.uni-lj.si (B.S.); brigita.tomsic@a.ntf.uni-lj.si (B.T.); 5 Faculty of Technology and Metallurgy, University of Belgrade, Karnegijeva 4, 11120 Belgrade, Serbia; maja@tmf.bg.ac.rs

**Keywords:** cotton, peat, chitosan, corona, copper oxide nanostructures

## Abstract

Growing demand for sustainable and green technologies has turned industries and research toward the more efficient utilization of natural and renewable resources. In an effort to tackle this issue, we developed an antibacterial textile nanocomposite material based on cotton and peat fibers with immobilized Cu-based nanostructures. In order to overcome poor wettability and affinity for Cu^2+^-ions, the substrate was activated by corona discharge and coated with the biopolymer chitosan before the in situ synthesis of nanostructures. Field emission scanning electron microscopy (FESEM) images show that the application of gallic or ascorbic acid as green reducing agents resulted in the formation of Cu-based nanosheets and mostly spherical nanoparticles, respectively. X-ray photoelectron spectroscopy (XPS) analysis revealed that the formed nanostructures consisted of Cu_2_O and CuO. A higher-concentration precursor solution led to higher copper content in the nanocomposites, independent of the reducing agent and chitosan deacetylation degree. Most of the synthesized nanocomposites provided maximum reduction of the bacteria *Escherichia coli* and *Staphylococcus aureus*. A combined modification using chitosan with a higher deacetylation degree, a 1 mM solution of CuSO_4_ solution, and gallic acid resulted in an optimal textile nanocomposite with strong antibacterial activity and moderate Cu^2+^-ion release in physiological solutions. Finally, the Cu-based nanostructures partially suppressed the biodegradation of the textile nanocomposite in soil.

## 1. Introduction

Global environmental concerns are continually forcing the development of eco-friendly and sustainable materials [1,2]. A vast amount of research has considered the fabrication of fibers and composites from natural and renewable resources, with the intention to reduce the unsustainable production of synthetic fibers and materials. Thanks to their specific properties, availability, low cost, biodegradability, and non-toxicity, natural fibers have been used in various industries, including for textiles, automobiles, packaging, sports, and medicines [1,3]. Furthermore, interest in natural and synthesized cellulose fibers is constantly growing. These fibers can be extracted from seeds (e.g., cotton, kapok, milkweed, and coir), stems (e.g., flax, hemp, jute, kenaf, and ramie), or leaves (e.g., sisal, abaca, and banana) [1,3]. To meet the increasing demand for natural cellulose fibers and sustainable industrial production, agricultural waste products or by-products, such as those associated with corn, wheat, rice, soybeans, and sugarcane, have been successfully exploited as sources of cellulose [2]. In addition, decomposed plants, such as cotton grass, which is a common plant in peat bogs, may also be utilized as a valuable source of cellulose fibers [3].

Peat is generated in flooded environments through the decomposition of various plant materials under acidic and anaerobic conditions [3,4]. It is commonly used for energy production, building, agriculture, infrastructure development, and medicine [5]. Peat fibers are produced from decomposed cotton grass stems extracted from the top layer of peat bogs, which is scrapped as waste. Thus, peat fibers are sustainable, as they are by-products of the peat excavation process and do not require additional agricultural land, water, or pesticides. Mikučioniene et al. reported that knits made of peat fibers mixed with cotton or wool fibers showed good features (e.g., abrasion resistance, heat transfer, and strength), and their utilization in the textile industry was recommended [3,5]. 

Despite its natural origin, the potential of peat fibers in manufacturing textile materials has not yet been sufficiently explored. Thus, we decided to investigate the possible application of a cotton/peat blend in the development of an antibacterial textile. Natural cellulose materials are prone to microbial attack, consequently inducing different structural and aesthetic changes, thus limiting the lifetime of the material and increasing the risk of human infection [6]. Metal and metal oxide (Ag, Cu, CuO, Cu_2_O, TiO_2_, and ZnO) nanoparticles (NPs) are excellent antimicrobial agents for textile finishing because small amounts of NPs can impart extraordinary antimicrobial activity to textiles [7,8,9]. In addition to Ag NPs that have been already commercially applied to medical textiles, many studies are dealing with the antimicrobial potential of ZnO NPs [10,11,12]. Recently, much attention has been paid to the impregnation of textiles with Cu-based NPs [9]. For this study, Cu-based NPs, as proven antimicrobial agents, were synthesized in situ on cotton/peat knit. To our knowledge, this is the first time that an antibacterial nanocomposite textile material composed of cotton and peat fibers has been successfully accomplished. The proposed procedure for the fabrication of the antibacterial textile nanocomposite consists of three steps: (i) fiber surface activation by corona discharge; (ii) coating with biopolymer chitosan; and (iii) the synthesis of Cu-based nanostructures by the sorption of Cu^2+^-ions, followed by their in situ reduction.

Preliminary studies revealed the necessity to hydrophilize the quite hydrophobic peat-based material before any further modification. This can be achieved through environmentally benign treatments with various forms of plasma operating at atmospheric or low pressures [13]. The ease of handling, a lack of a need for expensive vacuum pumps, and continuous processing suitable for textile mills were the reasons we chose corona discharge for the activation of the substrate in this study. A particular advantage of this treatment relies on the fact that plasma-induced oxidation (i.e., the production of new polar functional groups) is limited only to a thin layer of the fiber surface, leaving the bulk properties intact. 

In order to improve the uptake of Cu^2+^-ions by the corona-activated material, the biopolymer chitosan was employed. Chitosan is non-toxic, natural, cheap, biocompatible, and eco-friendly polymer with a high affinity for metal ions [14]. Keeping in mind that hydroxyl and particularly amino groups are the binding sites for Cu^2+^-ions in the chitosan chain, the influence of the deacetylation degree of chitosan on the final copper content was taken into consideration. In addition, the influence of the concentration of CuSO_4_ solution on the synthesis of Cu-based nanostructures was examined. The reduction of adsorbed Cu^2+^-ions, as a final step in the fabrication of the nanocomposites, was performed in situ on the substrate using gallic and ascorbic acids as green reducing agents. The developed nanocomposites were fully characterized using FESEM, Fourier-transform infrared spectroscopy (FTIR), atomic absorption spectroscopy (AAS), and X-ray Photoelectron Spectroscopy (XPS). XPS analysis provided excellent insight into the chemical structure of the nanocomposites and the chemical composition of synthesized nanostructures. The antibacterial activity of the immobilized nanostructures was tested against Gram-negative bacteria *E. coli* and Gram-positive bacteria *S. aureus*. The biodegradation of the developed nanocomposites was studied in soil under controlled humidity and temperature. 

## 2. Materials and Methods

### 2.1. Production of Peat-Based Material

The substrate was knitted in a single plain pattern on a circular one-bed weft knitting machine, Matec Techno New (Massa, Italy), with 3″ cylinder diameter and 14E gauge from blended cotton (75%)/peat (25%) yarns (linear density 60 tex × 1 × 4). In the following text, the obtained knitted fabric is denoted as CP. The chemical composition of the CP yarn, analyzed according to the Klason method [5] at the Latvian State Institute of Wood Chemistry, was 92.32% cellulose, 2.61% hemicellulose, 2.96% lignin, and 2.11% other compounds. The main characteristics of the knitted fabric were as follows: wale density, 4.2 cm^−1^; course density, 5.8 cm^−1^; loop length, 11.2 mm; wale spacing, 2.38 mm; course spacing, 1.72 mm; area density, 654.80 g/cm^2^; and tightness factor, 1.38 tex^1/2^/cm.

To remove the surface impurities, the substrate was immersed in a bath containing 0.05% non-ionic washing agent Felosan RG-N (CHT AG, Montlingen, Switzerland) at a liquid-to-fabric ratio of 40:1. The cleaning was performed at 60 °C for 30 min. The substrate was rinsed with tap and distilled water and then left to dry at room temperature.

### 2.2. Corona Treatment

CP samples were treated with corona discharge at atmospheric pressure using a commercial device (Vetaphone CP-Lab MK II, Kolding, Denmark). The samples were placed on an electrode roll covered with silicon coating, rotating at a minimum speed of 4 m/min. The power was set to 900 W, the number of passages to 30, and the distance between electrodes to 3.0 mm. In the following, corona-treated CP samples are denoted as CCP samples.

### 2.3. Preparation of Bio-Nanocomposites

#### 2.3.1. Coating of CCP Samples with Biopolymer Chitosan

For this study, two types of chitosan were used: chitosan with a deacetylation degree of 90% and viscosity of 847 mPa·s (CHT1), and chitosan with a deacetylation degree of 96% and viscosity of 102 mPa·s (CHT2). Both were supplied by Primex (Siglufrojdur, Iceland). A 1 *w*/*v*% CHT solution was prepared by dissolving 1.00 g of CHT in 100 mL of 1 *v*/*v*% acetic acid (Zorka Pharma, Sabac, Serbia), which was stirred at room temperature for 24 h. Afterwards, the solution was left to settle for 30 min in order to eliminate gas bubbles formed during agitation. 

Then, 1 g of CCP substrate was dipped into 30 mL of the freshly prepared CHT1 or CHT2 solutions. The samples were squeezed through a laboratory pad after 1 h and were dried at 80 °C for 10 min and after that, at room temperature. In the following, these samples are denoted as CCP-CHT1 and CCP-CHT2, respectively.

#### 2.3.2. Synthesis of Cu-Based Nanostructures

**Figure 1:**
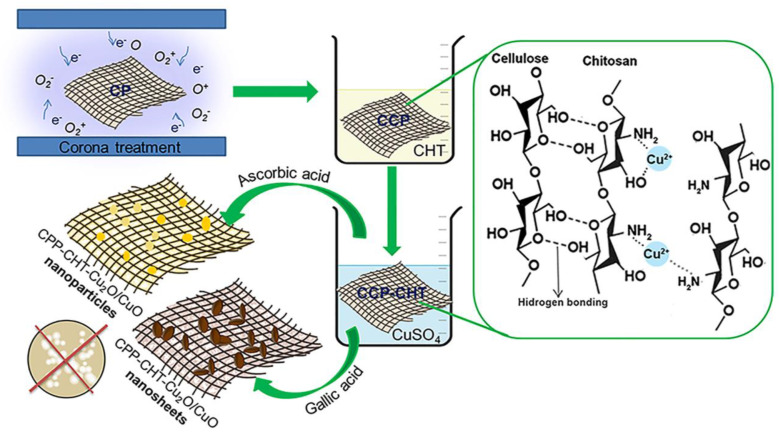
Schematic presentation of cotton/peat blend knit nano-finishing.

A total of 0.50 g of the CCP-CHT1 and CCP-CHT2 samples were immersed in 25 mL of CuSO_4_ (Centrohem, Stara Pazova, Serbia) solutions (1 mM, 5 mM, and 10 mM) for 2 h. The samples were then rinsed with distilled water, and the reduction of adsorbed Cu^2+^-ions was carried out in gallic and ascorbic acid solutions at 60 °C. The solution of gallic acid (Sigma-Aldrich, Merck KGaA, Darmstadt, Germany, purity ≥ 97%; GA) was prepared by dissolving 0.14 g of GA in a mixture of ethanol (1 mL) and distilled water (24 mL). The pH of the solution was adjusted to 4.50. For the other solution, 0.88 g of ascorbic acid (Centrohem, Stara Pazova, Serbia; AA) was dissolved in 25 mL of distilled water, and the pH was adjusted to 6.5. After reduction for 2 h, the samples were rinsed with distilled water and dried in air at room temperature. CCP-CHT1 and CCP-CHT2 samples loaded with 1 mM, 5 mM, or 10 mM CuSO4 solution and reduced with gallic acid are denoted as CCP-CHT1-Cu1-GA, CCP-CHT2-Cu1-GA, CCP-CHT1-Cu5-GA, CCP-CHT2-Cu5-GA, CCP-CHT1-Cu10-GA, and CCP-CHT2-Cu10-GA, respectively. In the same manner, the samples reduced with ascorbic acid are labeled as CCP-CHT1-Cu1-AA, CCP-CHT2-Cu1_AA, CCP-CHT1-Cu5-AA, CCP-CHT2-Cu5-AA, CCP-CHT1-Cu10-AA, and CCP-CHT2-Cu10-AA. A schematic illustration of the whole nano-finishing process is presented in Figure 1.

### 2.4. FTIR Analysis

FTIR spectra were collected over the range of 4000–600 cm^−1^ through 32 scans at a resolution of 4 cm^−1^ using a Spectrum 3 spectrophotometer (Perkin Elmer, Inc. Waltham, Massachusetts, USA) equipped with an attenuated total reflection (ATR) cell and a diamond crystal (*n* = 2.0). 

### 2.5. FESEM and NPs Size Analysis

The surface morphology of the fibers was evaluated using a FESEM (Mira3 Tescan, Tescan, Brno, Czech Republic). The samples were sputter-coated with a thin layer of Au before analysis. To define the size and size distribution of NPs, the obtained FESEM images were analyzed using the open-access imaging software tool ImageJ.

### 2.6. XPS Analysis

The chemical composition of the samples was assessed through XPS surface measurements. The C 1s, O 1s, N 1s, Cu 2p, and survey spectra were recorded using a Kratos Axis-Supra instrument (Kratos Analytical Ltd, Manchester, United Kingdom). A monochromatic X-ray source Al Kα (1486.6 eV) was used for all samples and experiments. The residual vacuum in the X-ray analysis chamber was maintained at around 4.9 × 10^−8^ Torr. The samples were fixed to the sample holder with double-sided carbon tape. Due to the non-conducting nature of the samples, it was necessary to use a co-axial electron neutralizer to minimize surface charging, which performed the neutralization by itself. Photoelectrons were collected from a take-off angle of 90° relative to the sample surface. The measurement was carried out in Constant Analyzer Energy mode (CAE) with a 15 mA emission current and 160 eV pass energy for survey spectra, and a 15 mA emission current and 20 eV pass energy for high-resolution spectra. Charge referencing was carried out by setting the binding energy of the C 1s photo peak at the 285.0 eV C 1s hydrocarbon peak. We also employed an electron flood gun to minimize surface charging (charge compensation). A wide-scan survey spectrum was used to identify and quantify the elements in the sample. High-resolution narrow scans were used to conduct the chemical state assessment, as well as to quantify the presence of reference elements in the sample. This process has an associated error of ± 0.1 eV. The CASAXPS software (version 2.3.15) was used to analyze spectra for elemental composition. Deconvolution into sub-peaks was performed using the least-squares peak analysis software XPSPEAK (version 4.1), using the Gaussian/Lorentzian sum function and Shirley-type background subtraction. In the peak fitting procedure, no tailing function was considered. The components of the various spectra were mainly modeled as symmetrical Gaussian peaks.

### 2.7. AAS Analysis

The total Cu content in nanocomposites was measured using atomic absorption spectrometry (AAS, Spectra AA 55 B, Varian, Mulgrave, Australia). Before the analysis, the samples were immersed in 1 V:1 V conc. HNO_3_:H_2_O (Zorka Pharma, Šabac, Serbia) solution for 24 h. The experiments were conducted in triplicate.

### 2.8. Antibacterial Test

The antibacterial activity of the Cu-based nanocomposites was evaluated against the Gram-negative bacteria *E. coli* ATCC 25922 and Gram-positive bacteria *S. aureus* ATCC 25923, according to the ASTM E 2149-01. Bacteria were cultivated in 3 mL of tryptone soy broth at 37 °C for 18 h (late exponential stage of growth). Then, 50 mL of sterile physiological saline solution was inoculated with 0.5 mL of bacterial inoculum. One gram of each sample with immobilized nanostructures was sterilized by autoclave (30 min) and put in a flask, which was shaken at 37 °C for 2 h. Aliquots from the flask were diluted with physiological saline solution and placed onto tryptone soy agar. After 24 h of incubation at 37 °C, zero-time (inoculum) and two-hour counts of viable micro-organisms were made. The percentage of bacterial reduction (*R*, %) was calculated in accordance with:

(1) $$R=\frac{C_{0}-C}{C_{0}}\cdot 100$$

where *C*_0_ (CFU/mL—colony forming units) is the number of bacteria colonies on the control CP sample and *C* (CFU/mL) is the number of bacteria colonies on the samples impregnated with Cu-based NPs.

### 2.9. Quantification of Cu^2+^-Ions Release

The release of Cu^2+^-ions was examined in accordance with the following procedure: samples impregnated with Cu-based NPs (0.25 g) were immersed into 25 mL of physiological saline solution (9 g/L NaCl) at 37 °C in static conditions. The concentration of released Cu^2+^-ions was measured after 1, 3, 6, and 24 h via AAS. The experiments were conducted in triplicate.

### 2.10. Biodegradation Test

The biodegradation of selected Cu-based nanocomposites was assessed using a soil burial test according to the ISO 11721-1: 2001 and ISO 11721: 2003 standards. A container filled with soil was sprayed with water during the test so that the soil water content of 60 ± 5% of its maximum moisture retention capacity remained constant. The pH of the soil ranged between 4.0 and 7.5. The knits were buried in the soil for 3, 9, and 18 days. After certain incubation times, the samples were removed from the soil, rinsed with running tap water, soaked in 70% ethanol for 30 min, and dried at room temperature.

## 3. Results and Discussion

### 3.1. Chemical and Morphological Characterization of the Samples

**Figure 2:**
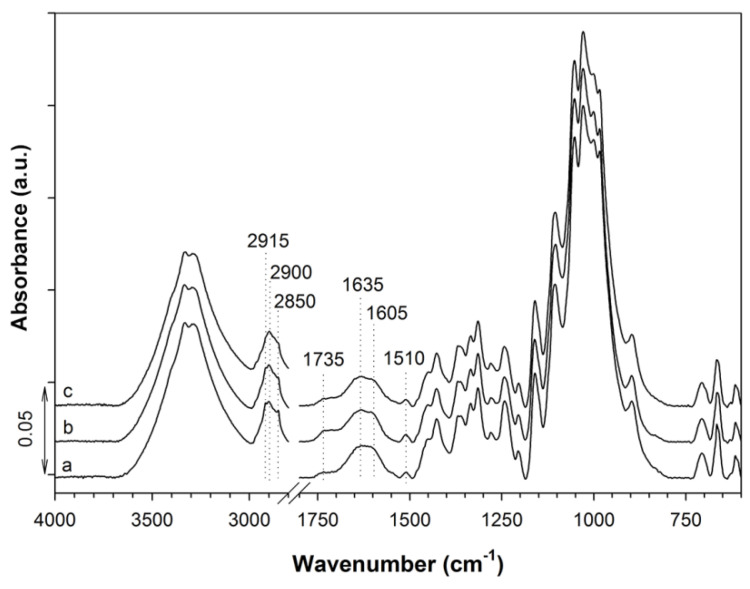
FTIR spectra of (a) CP; (b) CCP-CHT1; and (c) CCP-CHT2 samples.

FTIR spectra of CP, CCP-CHT1, and CCP-CHT2 samples are shown in Figure 2. In the spectrum of the CP sample, absorption bands attributed to the fingerprint of cellulose are clearly visible in the 1500–800 cm^−1^ spectral region, mainly related to C–O–C, C–H, C–O, and O–H vibrations. This was expected, as the CP yarn mainly consists of cellulose. In addition, an absorption band at 1640 cm^−1^, related to the vibration of water molecules, and an absorption band at 1735 cm^−1^, assigned to carboxyl groups, can be seen. The absorption band at 1510 cm^−1^, characteristic of C=C stretching in aromatic compounds, confirms the presence of lignin [15,16]. 

Preliminary studies indicated that the CP and CCP samples did not absorb sufficient amounts of Cu^2+^-ions to achieve the desired antibacterial activity. In our previous reports, carboxyl groups were imparted onto cotton fibers [17,18,19] to enhance the binding of Cu^2+^-ions. In addition to the carboxyl groups already present in the CP sample, amino groups from the chitosan coating were also introduced into the system, as they are also susceptible to complexation with Cu^2+^-ions. Accordingly, the presence of the CHT1 and CHT2 coatings on the CCP-CHT1 and CCP-CHT2 samples was confirmed by the formation of a shoulder at 1605 cm^−1^, which is characteristic of the C=O vibration of the amide group of chitosan. Moreover, the intensities of the absorption bands at 2915 cm^−1^ and 2850 cm^−1^ (assigned to C–H vibrations) were significantly decreased. This decrease was more pronounced in the spectrum of the CCP-CHT2 sample, which could be attributed to a higher degree of deacetylation of CHT2 (i.e., 96%), compared to that of CHT1 (i.e., 90%).

Considering that the amino group content has a significant impact on the amount of synthesized Cu-based nanostructures, the influence of the degree of deacetylation (i.e., 90% in CHT1 and 96% in CHT2) of chitosan on the total copper content was investigated in more detail (Table 1). In addition, the influence of the initial concentration of CuSO_4_ solution (1 mM, 5 mM, and 10 mM) on the total copper content in the nanocomposites was investigated (Table 1). The results in Table 1 clearly show that the total copper content was considerably more affected by the concentration of the precursor solution than by the applied reducing agent. The higher the concentration of the CuSO_4_ solution, the higher the copper content in the fabricated nanocomposites. There was no significant difference in copper content between the samples synthesized using the 5 mM and 10 mM solutions, regardless of the reducing agent used. In addition, the copper content in the CCP-CHT1-Cu5 and CCP-CHT2-Cu5 samples was evidently not affected by the type of chitosan and reducing agent. In the other cases, a slightly higher copper content was detected when the lower-viscosity chitosan solution (CHT2) was employed. This finding is in contrast with the recently reported results by Xiao et al. [6], who suggested that cotton fabric modified with chitosan of higher molecular weight and, thus, higher viscosity ensured higher copper content due to a stronger chelating ability. However, in the present study, the lower-viscosity chitosan (CHT2) had a higher deacetylation degree than the higher-viscosity chitosan (CHT1), indicating that more free amino groups were likely available for the binding of Cu^2+^-ions. 

**Figure 3:**
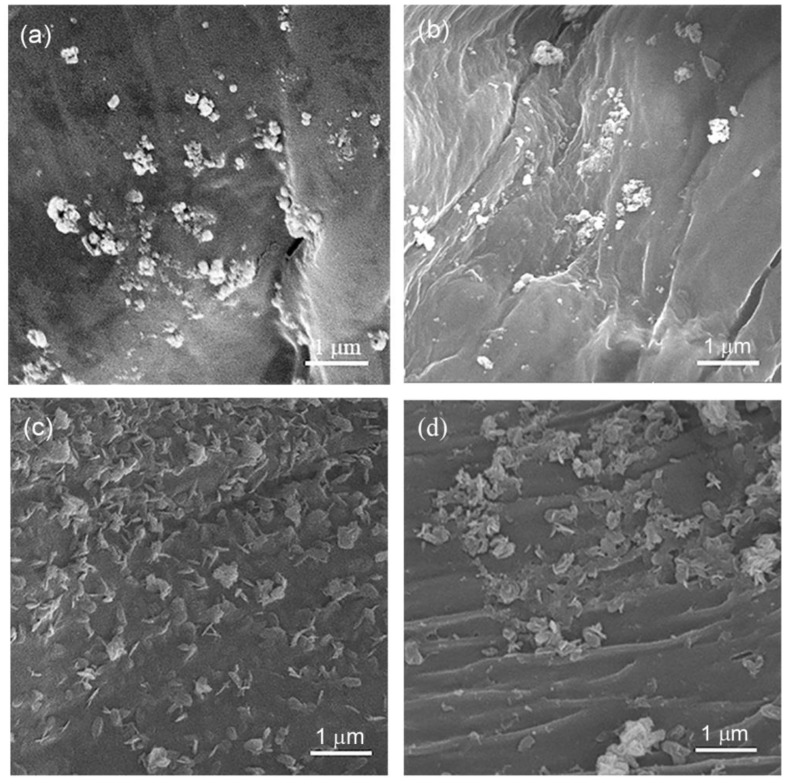
FESEM images of nanocomposites: (**a**) CCP-CHT1-Cu10-AA; (**b**) CCP-CHT2-Cu10-AA; (**c**) CCP-CHT1-Cu10-GA; and (**d**) CCP-CHT2-Cu10-GA.

**Figure 4:**
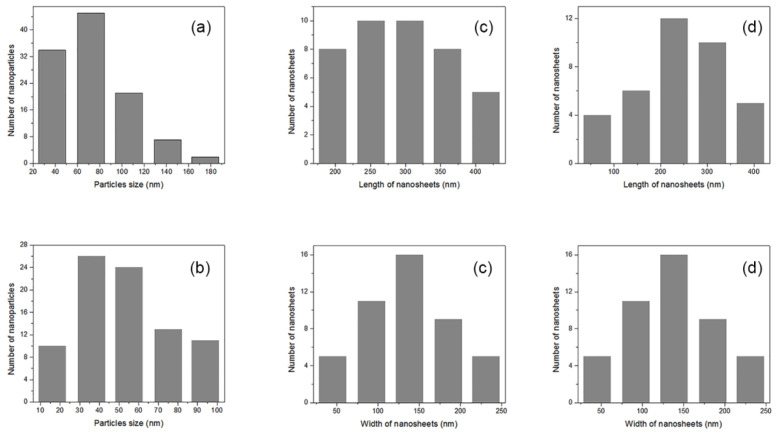
The size distribution of: (**a**) nanoparticles on CCP-CHT1-Cu10-AA; (**b**) nanoparticles on CCP-CHT2-Cu10-AA; (**c**) length and width of nanosheets on CCP-CHT1-Cu10-AA; and (**d**) length and width of nanosheets on CCP-CHT1-Cu10-AA.

The size, shape, and coating uniformity of Cu-based nanostructures synthesized on the surface of selected nanocomposites were analyzed using FESEM (Figure 3). Obviously, the shape and the size of the nanostructures were strongly dependent on the applied reducing agent. Spherical or nearly spherical NPs (Figure 3a,b) and nanosheets (Figure 3c,d) were formed when ascorbic and gallic acid, respectively, were used as reducing agents. Figure 3a,b reveal an uneven distribution of NPs across the CCP-CHT1-Cu10-AA and CCP-CHT2-Cu10-AA fibers, where larger amounts of single and smaller NPs are noticeable on the surface of CCP-CHT2-Cu10-AA fibers. The mean sizes of NPs synthesized on CCP-CHT1-Cu10-AA and CCP-CHT2-Cu10-AA fibers were 89 ± 31 nm and 63 ± 22 nm, respectively. The size distribution of NPs on these samples is presented in Figure 4a,b. Nanosheets synthesized on the surface of CCP-CHT1-Cu10-GA and CCP-CHT2-Cu10-GA fibers (Figure 3c,d) grew more uniformly, compared to NPs (Figure 3a,b). It is interesting that the surface of CP-CHT1-Cu10-GA fibers was covered only with nanosheets (Figure 3c), while both nanosheets and NPs were visible on the CP-CHT2-Cu10-GA fibers (Figure 3d). The mean length and width of nanosheets formed on CP-CHT1-Cu10-GA were 316 ± 54 nm and 166 ± 28 nm, respectively, and the size of nanosheets on CP-CHT2-Cu10-GA fibers was almost identical (165 ± 25 nm in width, 315 ± 42 nm in length). In addition, the mean size of NPs synthesized on CP-CHT2-Cu10-GA fibers was 67 ± 17 nm. The mean lengths of nanosheets synthesized on CP-CHT1-Cu10-GA and CP-CHT2-Cu10-GA fibers were similar, but the width and length distributions were quite different (Figure 4c,d). The share of shorter nanosheets was more pronounced on CP-CHT2-Cu10-GA fibers. 

Our recent results revealed that nanosheets synthesized with gallic acid on the surface of cotton fibers ranged from 377 to 723 nm in length and from 268 to 488 nm in width [17]. Evidently, the nanosheets synthesized in this study using the same reducing agent and following the same procedure were significantly smaller. Krkobabić et al. oxidized cotton fibers using a two-step process with NaIO_4_/NaClO_2_ prior to the synthesis of nanosheets, with the aim of introducing carboxyl groups as potential sites for complexation with Cu^2+^-ions [17]. In the current study, chitosan amino groups were employed for binding Cu^2+^-ions. It has been reported that metal cations could be attached as a pendant to the amino groups of chitosan [6,20,21,22]. Schlick et al. [23] suggested a bridge model based on the tetra-coordination of metal ions engaging nitrogen atoms from different chitosan chains when a square planar structure is formed [6,22,23]. Gomes et al. pointed out that Cu^2+^ ions can be bound to O(4) and N(5) in the glucosamine unit of chitosan, forming a c4 position of coordination [22]; hence, the difference in the size of the nanosheets may be a consequence of the different coordination of active groups with Cu^2+^-ions before the reduction process. 

**Figure 5:**
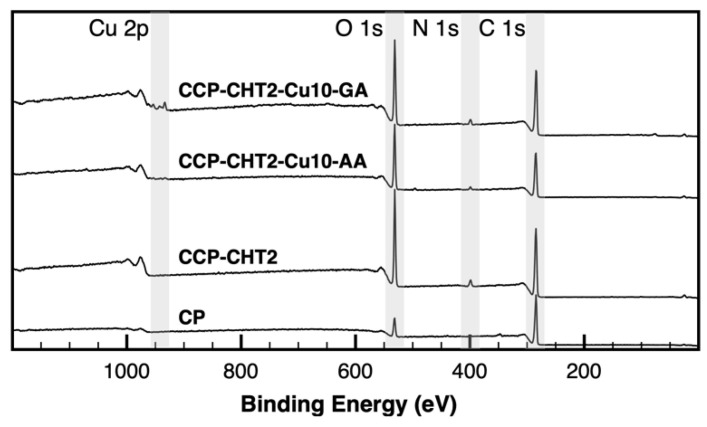
Survey XPS spectra of CP, CCP-CHT2, CCP-CHT2-Cu10-AA, and CCP-CHT2-Cu10-GA samples.

XPS analysis was performed on selected samples in order to determine the elements and the chemical bonds created during the synthesis of the nanocomposite materials. The survey XPS spectra were used to identify the major elements on the surfaces of CP, CCP-CHT2, CCP-CHT2-Cu10-AA, and CCP-CHT2-Cu10-GA samples (Figure 5). Peaks in the regions attributed to carbon (C 1s), oxygen (O 1s), and nitrogen (N 1s) were identified in the spectra of CP and CCP-CHT2 samples. Corona treatment and the addition of chitosan caused a significant increase in the percentages of oxygen (from 12.11 ± 0.25% to 27.68 ± 0.29%) and nitrogen (from 1.62 ± 0.32% to 4.39 ± 0.24%); see Table 2. Such a trend was expected, as the functionalization of materials with oxygen-activated groups using air corona treatment has been widely reported [24,25]. Additionally, the hydroxyl and amino groups of chitosan on each unit also contributed to the increases in oxygen and nitrogen content [26]. An equivalent trend was observed in the O/C and N/C ratios, where the values significantly increased in the CCP-CHT2 sample (Table 2). As expected, peaks corresponding to copper were only detected in the CCP-CHT2-Cu10-AA and CCP-CHT2-Cu10-GA samples (Figure 5). The elemental identification analysis suggested that the atomic percentages of Cu 2p were comparable, indicating a similar immobilization of Cu-based nanostructures on the fiber surfaces, independent of the applied reducing agent (Table 2). 

**Figure 6:**
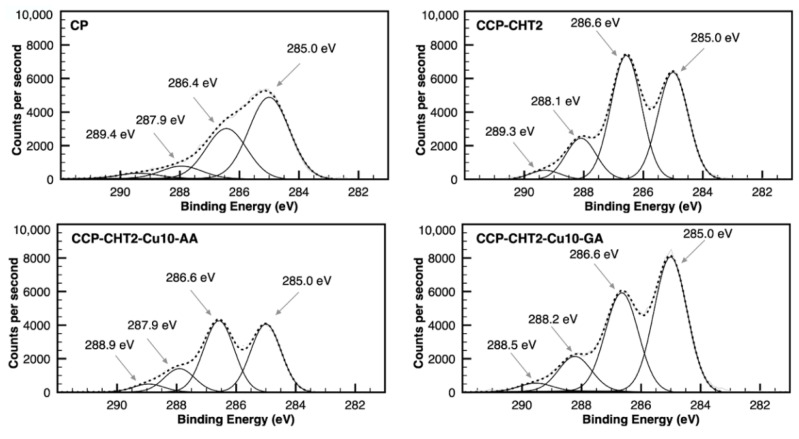
Gaussian deconvolution of high-resolution XPS C 1s spectra for CP, CCP-CHT2, CCP-CHT2-Cu10-AA, and CCP-CHT2-Cu10-GA samples.

High-resolution spectra for C 1s, O 1s, N 1s, and Cu 2p were collected in order to understand the different chemical bonds on the surfaces of the samples, as well as to define the oxidation state of the Cu-based nanostructures. Considering the fabric composition (92.32% cellulose, 2.61% hemicellulose, 2.96% lignin, and 2.11% other compounds), the XPS deconvolutions of the C 1s spectra in the CP sample showed four carbon peaks commonly found in cellulose materials. The peaks at 285.0 eV, 286.4 eV, and 287.9 eV were attributed to the C–C/C–H, C–O, and C=O/O–C–O groups, respectively (Figure 6). The additional peak at 289.4 eV may correspond to O–C=O groups in lignin and in the other 2.11% unidentified compounds [27,28,29,30]. The C 1s high-resolution spectra of the CCP-CHT2 sample revealed more intense peaks at 286.6 eV and 287.9–288.2 eV, due to the addition of C–N and C–O groups through chitosan [31]. The relative peak areas of C–NH or C–NH_2_ bonds significantly decreased in the CCP-CHT2-Cu10-AA and CCP-CHT2-Cu10-GA nanocomposites, compared to the CP-CHT2 sample. This may indicate some degree of surface functionality changes (e.g., fewer free amino groups) on the chitosan coating surface, likely due to coordination with copper nanoparticles [32,33]. 

**Figure 7:**
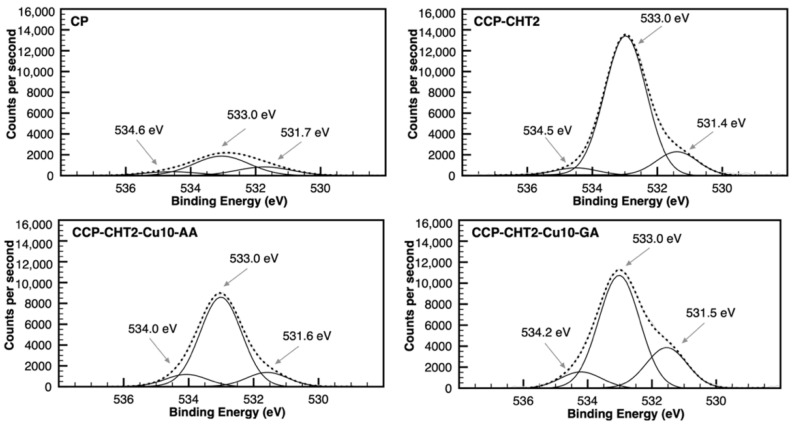
Gaussian deconvolution of high-resolution XPS O 1s spectra from CP, CCP-CHT2, CCP-CHT2-Cu10-AA, and CCP-CHT2-Cu10-GA samples.

The O 1s deconvolution spectra exhibited three fitting peaks at 531.4–531.7 eV, 533.0 eV, and 534.1–534.6 eV (Figure 7). The major peak at 533.0 eV corresponded to the single-bonded oxygen in alcohol groups present in cellulose, chitosan, and lignin. Next, double-bonded oxygen (i.e., carbonyl and carboxyl groups) was associated with lignin [34,35,36]. The minor peak at 534.1–534.6 eV was attributed to possible carboxyl groups present in other components of the fibers [37,38].

**Figure 8:**
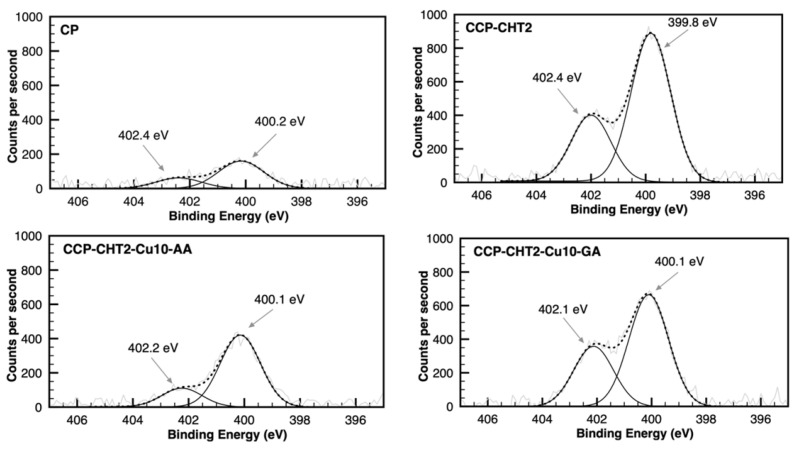
Gaussian deconvolution of high-resolution XPS N 1s spectra from CP, CCP-CHT2, CCP-CHT2-Cu10-AA, and CCP-CHT2-Cu10-GA samples.

The N 1s spectra of the samples were deconvoluted into two peaks with binding energies at 399.8–400.2 eV and 402.1–402.4 eV, which were assigned to amine and protonated amine species in chitosan, respectively (Figure 8) [39]. The intensity of the peaks in the N 1s high-resolution spectra for the CP sample was very low, compared to that in other samples (also supported by the elemental percentages provided by survey spectra), suggesting very few nitrogen-containing groups in the unidentified compounds present in the CP sample. A slightly decreased peak for C–NH_2_ groups was observed in CCP-CHT2-Cu10-AA and CCP-CHT2-Cu10-GA, compared to the CCP-CHT sample, in accordance with the C 1s high-resolution spectra. 

**Figure 9:**
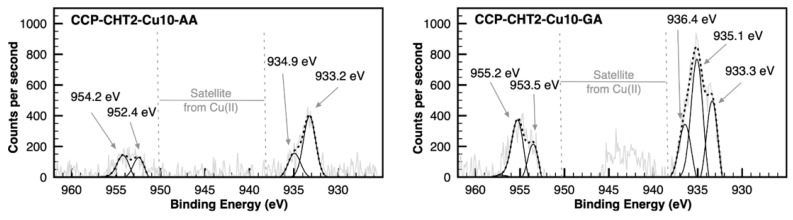
Gaussian deconvolution of high-resolution XPS Cu 2p spectra CCP-CHT2-Cu10-AA and CCP-CHT2-Cu10-GA samples.

The Gaussian deconvolution of Cu 2p high-resolution XPS spectra indicated the coexistence of CuO and Cu_2_O in both CCP-CHT2-Cu10-AA and CCP-CHT2-Cu10-GA samples. An additional peak was observed in the CP-CHT2-Cu10-GA spectrum, which was ascribed to CuSO_4_ salt (Figure 9 and Table 3). The Cu 2p3/2 region in the CCP-CHT2-Cu10-AA sample could be deconvoluted into two peaks at 933.2 eV and 934.9 eV. The Cu 2p1/2 region was also deconvoluted into two peaks at 952.4 eV and 954.2 eV. The peaks at 933.2 eV and 952.4 eV were assigned to the Cu^+^-ions of oxidized species, while the peaks at 934.9 eV and 954.2 eV indicated the Cu^2+^-ions of oxidized species [40,41]. Similarly, the same copper species were observed on the surface of the CCP-CHT2-Cu10-GA sample, through the emergence of two peaks at the binding energies corresponding to Cu 2p3/2. However, the peaks in the Cu 2p1/2 suffered a slight deviation, occurring at 953.5 eV and 955.2 eV. These peaks were also ascribed to Cu_2_O and CuO species, respectively [40,41,42]. In addition, the presence of satellite peaks of Cu 2p spectra could be clearly observed in the CCP-CHT2-Cu10-GA sample, revealing the Cu^2+^-ions of oxidized species on the sample surface [35]. To the contrary, the peak corresponding to CuO (~935 eV) in the CCP-CHT2-Cu10-AA sample was much lower than that in the CCP-CHT2-Cu10-GA sample and, consequently, the respective satellites did not clearly appear. The additional peak in the CCP-CHT2-Cu10-GA sample at 936.4 eV can be commonly attributed to Cu^2+^-ions present in CuSO_4_ salts, suggesting an incomplete salt reduction when using gallic acid [43]. 

### 3.2. Antimicrobial Activity

The major goal of this study was to fabricate CP textile nanocomposites with antimicrobial activity. It is believed that the antimicrobial action of Cu-based nanostructures relies on several different mechanisms, including attack by Cu^2+^-ions, the Cu-mediated generation of reactive oxygen species, and direct contact with nanomaterials [44,45,46,47]. Some authors have suggested that Cu^2+^-ions released from NPs react with the negatively charged bacterial cell wall, inducing protein denaturation and cell death [44]. Accordingly, we expected that the in situ synthesized Cu_2_O/CuO NPs and nanosheets could inhibit the growth of micro-organisms, thus prolonging the lifetime of nanocomposites. Hence, the antimicrobial activity of the novel nanocomposites was tested against the Gram-negative bacteria *E. coli* and Gram-positive bacteria *S. aureus*. The observed antibacterial activities imparted to the nanocomposites are summarized in Table 4. Apparently, CP, CCP-CHT1, and CCP-CHT2 samples did not provide any antibacterial activity, while all samples reduced with gallic acid exhibited a maximum level of antibacterial activity (R = 99.9%), indicating that Cu_2_O/CuO nanosheets act as a very strong antibacterial agent. It is important to stress that the synthesized nanosheets ensured excellent antibacterial activity independently of their copper content (Table 4). At the same time, the total copper content (Table 1) did not significantly affect the samples reduced with ascorbic acid, which provided maximum antibacterial activity against *S. aureus* (R = 99.9%). The influence of copper content was more prominent in the case of antibacterial action against bacteria *E. coli*: only the samples synthesized using 5 mM and 10 mM CuSO_4_ solutions provided the maximum reduction of *E. coli* (R = 99.9%), due to their higher copper content (Table 1). The poor antibacterial activities of CCP-CHT1-Cu1-AA (R = 86.8%) and CCP-CHT2-Cu1-AA (R = 89.8%) samples against *E. coli* are likely due to the lower copper content in these samples. Still, these amounts were found to be sufficient to impart bacteriostatic activity. 

Taking into consideration the results presented in Table 1 and Table 4 and Figure 3, it can be concluded that the total amount of copper and the shape of the synthesized nanostructures have strong impacts on the antibacterial activity of the synthesized nanocomposites, which is in line with existing data from the literature [48,49]. 

### 3.3. Release of Cu^2+^-Ions

**Figure 10:**
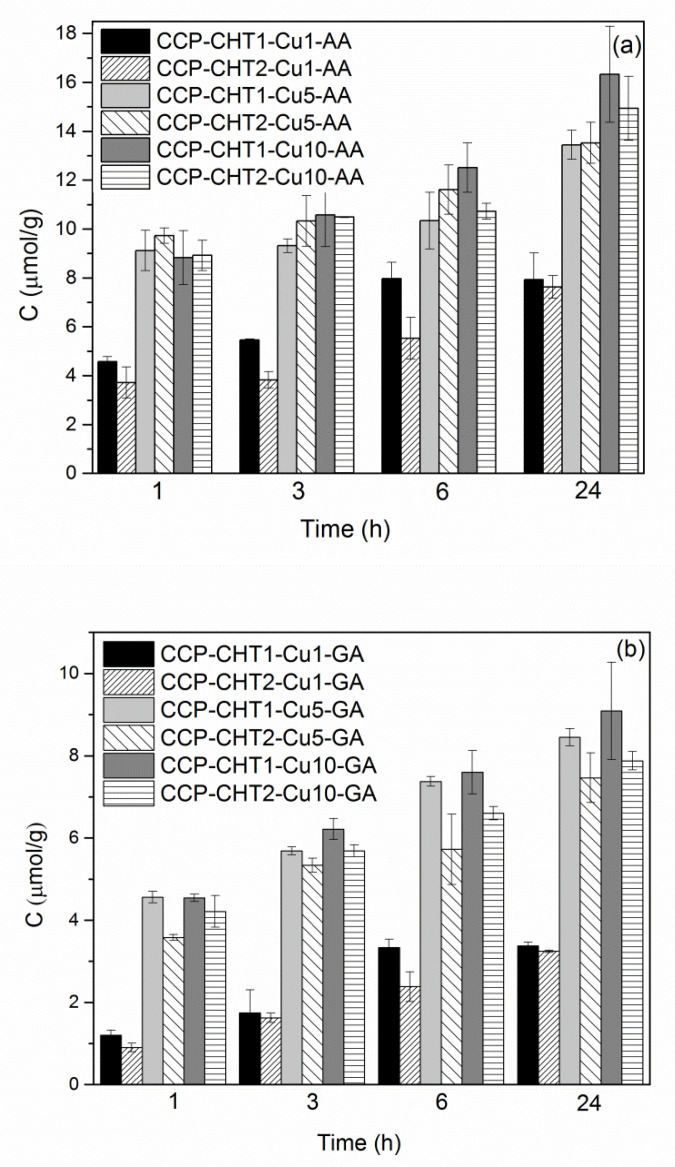
Release of Cu^2+^-ions into physiological saline solution from samples reduced with: (**a**) ascorbic acid; and (**b**) gallic acid.

It is important to investigate the behavior of nanocomposites in physiological systems, as the release of Cu^2+^-ions from the samples into the surrounding medium could be related to the cytotoxicity of the synthesized nanomaterials [50,51]. Hence, physiological saline solution was chosen as a release medium. The cumulative release of Cu^2+^ ions from the nanocomposites in physiological saline solution within 24 h is presented in Figure 10. Evidently, the leaching of Cu^2+^-ions strongly depended on the applied reducing agent, the total copper content in the nanocomposite, and the viscosity of the chitosan solution. Larger amounts of Cu^2+^-ions were released from the samples reduced using ascorbic acid. Furthermore, larger amounts of Cu^2+^-ions were leached from samples with higher copper content, independent of the applied reducing agent. Significantly lower amounts of Cu^2+^-ions were released from the samples synthesized using the 1 mM solution, compared to 5 mM and 10 mM solutions of the precursor salt. A reduced release of ions was detected for all samples modified, with the chitosan solution having lower viscosity. Depending on the total copper content, the maximum amounts of released Cu^2+^-ions originating from the nanocomposites reduced with gallic or ascorbic acid varied between 3.2–9.1 μmol/g and 7.6–16 μmol/g. Our previous studies indicated that Cu-based nanosheets and NPs obtained by reduction using gallic or ascorbic acid on cotton and PP fabrics were not cytotoxic to HaCaT cells in concentrations lower than 16 μmol/g and 10 μmol/g, respectively [17,49]. Accordingly, all nanocomposites impregnated with nanosheets can be considered safe for use in contact with human skin. In contrast, only the nanocomposites with the lowest amounts of immobilized NPs are potentially non-cytotoxic to human skin cells. Similar cytotoxicity threshold levels have been found in several previous reports [52,53,54,55]. In addition to the concentration, nanomaterial size and surface functionalization also have an influence on the cytotoxicity of Cu-based nanostructures, which has been discussed in the literature [50,55,56]. 

### 3.4. Biodegradation Behavior of the Samples in Soil

**Figure 11:**
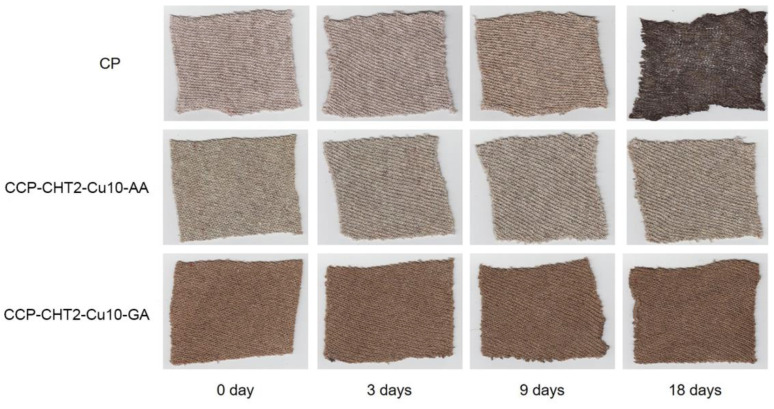
Photographs of studied samples before (0 days) and after 3, 9, and 18 days of soil burial (the sample size: 10 cm × 8 cm).

**Figure 12:**
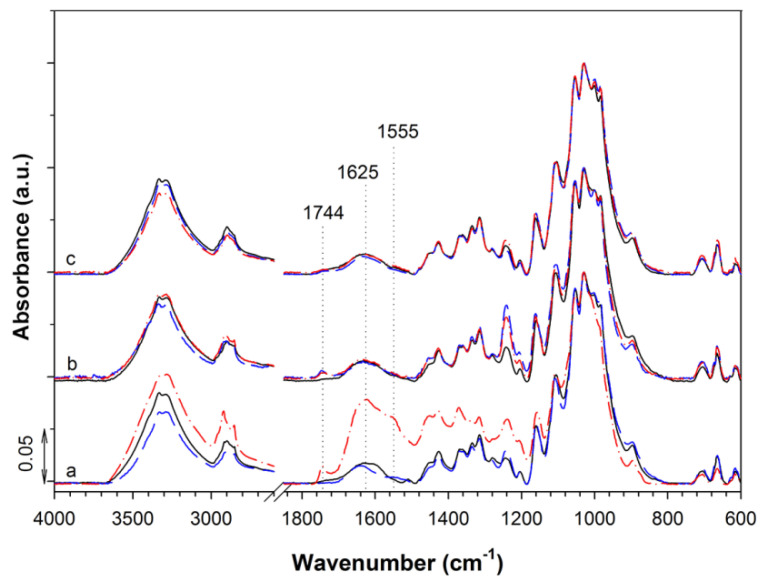
IR ATR spectra of: (a) CP; (b) CCP-CHT2-Cu10-AA; and (c) CCP-CHT2-Cu10-GA samples before (black full line) and after 9 (blue dash line) and 18 (red dash-dot line) days of soil burial.

The biodegradation behavior of the studied samples was investigated using a soil burial test in order to obtain information on the influence of the modification process on the degradation rate in the soil, which is an important parameter when the modified products are disposed of and are no longer in use. In addition, this in vitro analysis also provided information on the antimicrobial activity of the samples, not only for a single bacterial strain, but for a mixture of different micro-organisms present in the humus soil. Accordingly, the studied CP, CCP-CHT2-Cu10-AA, and CCP-CHT2-Cu10-GA samples were buried in model humus soil for a period of 18 days and were additionally observed at 3 and 9 days. The results are shown in Figure 11 and Figure 12. The control CP sample showed relatively high resistance to soil microflora until 9 days after burial. However, after 18 days of exposure to soil microflora, the CP sample showed strong color changes towards dark brown and black shades (Figure 11), due to rotting of the CP fibers. On the other hand, the CCP-CHT2-Cu10-AA and CCP-CHT2-Cu10-GA samples did not show a significant color change even at the end of the whole burial period, indicating high resistance to the complex structure of soil micro-organisms and confirming the high antimicrobial activity of the applied Cu-based nanostructures.

The chemical changes which occurred in the CP, CCP-CHT2-Cu10-AA, and CCP-CHT2-Cu10-GA samples during the burial period were investigated using FTIR analysis (Figure 12). The high susceptibility of the CP sample to soil micro-organisms from the 9th to 18th days of burial in humus soil was confirmed by the formation of high-intensity absorption bands at 1625 and 1555 cm^−1^, assigned to amide I and amide II of secondary polyamides present in proteins produced by micro-organisms [57,58]. This undoubtedly confirms the intensive growth of micro-organisms and the formation of biofilms on the surfaces of the CP fibers. Moreover, the increase in the intensity of the absorption band at 1744 cm^−1^, due to aldehyde and carboxyl groups, indicates the hydrolysis and strong oxidative degradation of the CP sample during the biodegradation process. Such intense chemical changes were not detected in the spectra of the CCP-CHT2-Cu10-AA and CCP-CHT2-Cu10-GA samples, which is consistent with the color change of these two samples after burial. However, a closer examination of the CCP-CHT2-Cu10-AA spectra after 9 and 18 days of burial showed an increase in the intensity of the absorption band at 1744 cm^−1^, accompanied by a sharp increase in the absorption band at 1245 cm^−1^, indicating some susceptibility of the CCP-CHT2-Cu10-AA sample to biodegradation. This could be due to a higher release of Cu^2+^-ions and, thus, more intense leaching of the Cu-based nanostructures from the surface of the CCP-CHT2-Cu10-AA sample to the soil, creating slightly better conditions for microbial growth, when compared to the surface of the CCP-CHT2-Cu10-GA sample. According to this assumption, the application of Cu-based nanostructures does not completely hinder the biodegradation process, but only slows it down, which is consistent with our previous study [59]. 

## 4. Conclusions

Antibacterial nanocomposite materials were successfully produced using a corona-discharge-activated knitted fabric made of a cotton/peat blend covered with the biopolymer chitosan and Cu_2_O/CuO nanostructures. Increases in the O/C and N/C ratios, as quantified by XPS analysis, confirmed the efficient activation of the samples after corona discharge treatment and chitosan coating. FESEM analysis revealed that the application of ascorbic or gallic acid as green reducing agents resulted in the formation of spherical nanoparticles (~63 nm) and nanosheets (~315 nm × 165 nm) on the fiber surfaces, respectively. XPS analysis indicated that the synthesized nanostructures were a mixture of Cu_2_O and CuO. The total copper content after the synthesis of the nanostructures was strongly affected by the concentration of CuSO_4_, the deacetylation degree of chitosan, and the applied reducing agent. The application of gallic acid in the reduction step provided maximum antibacterial activity against *E. coli* and *S. aureus*, independent of the initial concentration of the precursor salt and the acetylation degree of chitosan. In the case of ascorbic acid, such results were only obtained with nanocomposites synthesized using 5 and 10 mM precursor solutions. The overall cumulative release of Cu^2+^-ions in the physiological saline solution varied with the total copper content, the employed reducing agent, and the viscosity of the chitosan solution. The biodegradation test revealed that the control knitted fabric degraded faster in soil, indicating the inhibitory role of the Cu-based nanostructures. Taking into consideration the antibacterial activity, Cu^2+^-ions release, and the potential consequent cytotoxicity of the nanocomposites, optimum results can be obtained by employing biopolymer chitosan with a higher deacetylation degree, a 1 mM solution of CuSO_4_, and gallic acid as a reducing agent.

## Figures and Tables

**Table 1:** Total copper content (C) in nanocomposites.

Sample	C (µmol/g)	
	Reduced with GA	Reduced with AA
CCP-CHT1-Cu1	28 ± 1.7	24 ± 0.3
CCP-CHT2-Cu1	32 ± 2.3	25 ± 0.8
CCP-CHT1-Cu5	48 ± 0.2	48 ± 0.7
CCP-CHT2-Cu5	48 ± 0.2	48 ± 1.0
CCP-CHT1-Cu10	51 ± 2.8	50 ± 2.9
CCP-CHT2-Cu10	54 ± 2.8	55 ± 3.9

**Table 2:** Relative chemical composition (at.%) and atomic ratios on the surface of CP, CCP-CHT2, CCP-CHT2-Cu10-AA, and CCP-CHT2-Cu10-GA samples derived from the XPS survey spectra.

Samples	C 1s (at.%)	O 1s (at.%)	N 1s (at.%)	Cu 2p (at.%)	O/C Ratio	N/C Ratio
CP	85.86 ± 0.41	12.11 ± 0.25	1.62 ± 0.32	-	0.14	0.02
CCP-CHT2	67.62 ± 0.34	27.68 ± 0.29	4.39 ± 0.24	-	0.41	0.06
CCP-CHT2-Cu10-AA	65.34 ± 0.45	30.62 ± 0.40	2.85 ± 0.26	1.20 ± 0.17	0.47	0.04
CCP-CHT2-Cu10-GA	67.95 ± 0.38	27.13 ± 0.33	3.64 ± 0.24	1.28 ± 0.11	0.40	0.05

**Table 3:** Results of the XPS deconvolution analysis of the Cu 2p peaks for CCP-CHT2-Cu10-AA and CCP-CHT2-Cu10-GA reported as relative area corresponding to different chemical bonds (%).

Samples	Cu_2_O	CuO	CuSO_4_	Cu_2_O	Cu_2_O	CuO	CuO
	933.2 eV	935.0 eV	936.4 eV	952.4 eV	953.5 eV	954.2 eV	955.2 eV
CCP-CHT2-Cu10-AA	46.2%	17.9%	-	18.9%	-	17.1%	-
CCP-CHT2-Cu10-GA	25.1%	32.7%	14.0%	-	12.6%	-	15.6%

**Table 4:** Antimicrobial activity of investigated nanocomposites.

Sample	Microorganisms (CFU/mL)	
	*E. coli*	*S. aureus*
Inoculum	5.6 × 10^6^	2.5 × 10^6^
CP	4.1 × 10^6^	9.0 × 10^5^
CCP-CHT1	7.0 × 10^6^	3.0 × 10^5^
CCP-CHT1-GA	3.5 × 10^6^	2.5 × 10^5^
CCP-CHT1-AA	4.2 × 10^6^	3.4 × 10^5^
CCP-CHT2	1.2 × 10^6^	3.0 × 10^5^
CCP-CHT2-GA	1.3 × 10^6^	3.1 × 10^5^
CCP-CHT2-AA	2.8 × 10^6^	8.0 × 10^5^
CCP-CHT1-Cu1-GA	<10	<10
CCP-CHT2-Cu1-GA	<10	<10
CCP-CHT1-Cu5-GA	<10	<10
CCP-CHT2-Cu5-GA	<10	<10
CCP-CHT1-Cu10-GA	10	<10
CCP-CHT2-Cu10-GA	<10	<10
CCP-CHT1-Cu1-AA	5.4 × 10^5^	10
CCP-CHT2-Cu1-AA	4.2 × 10^5^	200
CCP-CHT1-Cu5-AA	110	<10
CCP-CHT2-Cu5-AA	20	<10
CCP-CHT1-Cu10-AA	<10	<10
CCP-CHT2-Cu10-AA	<10	<10

## Data Availability

Not applicable.
